# Surfactant Protein-A Function: Knowledge Gained From SP-A Knockout Mice

**DOI:** 10.3389/fped.2021.799693

**Published:** 2022-01-07

**Authors:** Lynnlee Depicolzuane, David S. Phelps, Joanna Floros

**Affiliations:** ^1^Departments of Pediatrics, Hershey, PA, United States; ^2^Obstetrics and Gynecology, The Pennsylvania State University College of Medicine, Hershey, PA, United States

**Keywords:** surfactant, host defense, SP-A, infection, injury, animal model, respiratory distress syndrome, innate immunity

## Abstract

Pulmonary surfactant proteins have many roles in surfactant- related functions and innate immunity. One of these proteins is the surfactant protein A (SP-A) that plays a role in both surfactant-related processes and host defense and is the focus in this review. SP-A interacts with the sentinel host defense cell in the alveolus, the alveolar macrophage (AM), to modulate its function and expression profile under various conditions, as well as other alveolar epithelial cells such as the Type II cell. Via these interactions, SP-A has an impact on the alveolar microenvironment. SP-A is also important for surfactant structure and function. Much of what is understood of the function of SP-A and its various roles in lung health has been learned from SP-A knockout (KO) mouse experiments, as reviewed here. A vast majority of this work has been done with infection models that are bacterial, viral, and fungal in nature. Other models have also been used, including those of bleomycin-induced lung injury and ozone-induced oxidative stress either alone or in combination with an infectious agent, bone marrow transplantation, and other. In addition, models investigating the effects of SP-A on surfactant components or surfactant structure have contributed important information. SP-A also appears to play a role in pathways involved in sex differences in response to infection and/or oxidative stress, as well as at baseline conditions. To date, this is the first review to provide a comprehensive report of the functions of SP-A as learned through KO mice.

## Introduction

Pulmonary surfactant has many critical roles in respiratory function. One is to decrease surface tension at the air-liquid interface, preventing alveolar lung collapse at low lung volumes. The administration of exogenous surfactant containing surfactant proteins B and C (SP-B and SP-C) is used routinely to treat prematurely born infants at risk for respiratory distress syndrome (RDS) (1). Pulmonary surfactant is a lipoprotein complex consisting of lipids, primarily phospholipids, and several proteins, SP-A, SP-B, SP-C and SP-D (the latter co-isolates with surfactant). These proteins collectively play various roles in surfactant-related functions and innate immunity/host defense (2). The focus of this review is on SP-A actions as learned from studies of SP-A knockout (KO) mice.

SP-A has been shown to be involved in surfactant-related function or structure and in the regulation of inflammatory processes and innate host defense. SP-A interacts in the alveolar space with the sentinel resident cell in alveolar host defense, the alveolar macrophage (AM). Via its interaction with the AM, SP-A affects the status and functions of AM under baseline conditions or in response to various insults. In addition, SP-A either via its interaction with the AM or other cells in the alveolus, imparts changes in the alveolar microenvironment where the AM resides that, in turn, may have an impact on AM baseline status and its overall activity in response to various insults (2).

Humans have two *SFTPA* genes and these have been identified with extensive genetic and epigenetic variability and human SP-A protein variants have been shown to differentially affect AM and alveolar epithelial type II cells, as well as the alveolar microenvironment. These differential effects have been observed both under baseline conditions and in response to various insults, as assessed by studies of bronchoalveolar lavage samples, alveolar cells, or other. However, the differential effects of the human SP-A protein variants have been reviewed recently elsewhere and will not be discussed here (2). The specific focus of this review is on knowledge gained, with regards to SP-A function, via comparison studies of SP-A KO and wild type (WT) mice or KO mice expressing an SP-A transgene. The direct and indirect roles of SP-A in combating various bacterial and viral pathogens or other types of insults, as well as the various SP-A-mediated activities that may affect surfactant structure, or activities of various surfactant components are noted.

## Bacterial Pathogens

Following the generation of SP-A KO mice via gene targeting techniques, the initial studies showed that these mice survived and bred normally under controlled housing conditions. Lung morphology and characteristics of surfactant and its components largely were not much different from WT mice, other than a scarcity of tubular myelin, an extracellular structural form of surfactant, and the observation that higher minimum surface tension was observed at low surfactant concentrations in KO (3). However, bacterial clearance in the KO was less efficient, pointing to a role for SP-A in innate immunity (4). Since then, infection studies with a multitude of bacterial strains have been examined to gain insight into the role of SP-A in host defense. The evidence from these studies indicates that the involvement of SP-A has a positive role in innate immune response.

The alveolar macrophage serves as the primary phagocyte of the innate immune system in the lung. Many of the interactions of SP-A involve this important population of cells. In a study of *Staphylococcus aureus* infection, SP-A has been found to bind to the C1q receptor on monocytes (5), as well as to interact with SP-R210 on AM (6). SP-A-opsonized bacteria are shown to lead to macrophage activation and secretion of tumor necrosis factor (TNF)-alpha (5, 6). Moreover, the *S. aureus* extracellular adherence protein (Eap) is a critical protein ligand for SP-A-mediated binding to *S. aureus* and necessary for the *in vivo* clearance of acute *S. aureus* infection (7). Veith et al. showed that in an *S. aureus* infection model, SP-A KO mice compared to WT showed a significantly lower number of phagocytes, mostly AM, filled with staphylococci at early post-infection intervals (8).

In infection with *Escherichia coli*, there has been evidence that SP-A enhances the binding and deacylation of *E. coli* lipopolysaccharide (LPS) by AM (9). In an infection model with *non-typeable Haemophilus influenzae* or *Group B streptococcus* infection, SP-A KO mice, in comparison to WT, showed a decrease in bacterial clearance, decreased association and phagocytosis of bacteria, as well as decreased superoxide and hydrogen peroxide production by AM. Increased lung pathology and increased total cells and polymorphonuclear neutrophils (PMNs) in bronchoalveolar lavage (BAL) were observed as well as an increase in levels of pro-inflammatory cytokines, TNF-α, interleukin 1 beta (IL-1β), interleukin 6 (IL-6), and macrophage inflammatory protein (MIP)-2 in lung homogenates (10). LeVine et al. had also conducted prior studies looking at a *Group B streptococcus* infection model. In one study, SP-A KO mice compared to WT showed an increase in pulmonary infiltration at 6 and 24 h post-infection, increased number of bacteria in lung homogenates, increased dissemination of bacteria to the spleen, and decreased association of bacteria with AMs (11). Another study by this group showed that in SP-A KO mice compared to WT, there was a decreased phagocytosis of bacteria by macrophages, decreased superoxide production in BAL, and rescue by exogenous SP-A increased clearance of bacteria (12).

In a *Pseudomonas aeruginosa* infection model, in SP-A KO mice compared to WT, there was a decreased clearance of bacteria, increased lung inflammation and bacterial burden in lungs, and decreased phagocytosis of bacteria by AMs (13, 14). In one study the SPA-4 peptide, derived from the C-terminal TLR-4 interacting region of SP-A, was administered *in P. aeruginosa* infected mice, and the pro-phagocytic and anti-inflammatory activity of the SPA-4 peptide led to reduced bacterial burden and decreased levels of cytokines and chemokines (15).

Other data indicate a large impact on the overall host defense system by SP-A. Ali et al. showed in their *K. pneumoniae* infection model that in the SP-A KO mice compared to WT ~75% of 32 host defense proteins were lower in BAL of uninfected mice, indicating that the uninfected mice hold the potential for increased susceptibility to infection. However, higher levels of more than two-thirds of the identified proteins at 4 h post-infection were observed in KO vs. WT, which was almost the exact opposite of the untreated mice (16), indicating, perhaps, an attempt by the KO to compensate for its baseline deficits. However, in terms of the significant changes in protein levels, more significant changes were observed in WT infected compared to KO infected. In an otitis media model of infection with *non-typeable Haemophilus influenzae*, SP-A was shown to modulate the expression of pro-inflammatory markers, IL-6 and IL-1β, and these peaked at higher levels in SP-A KO mice (17).

## Sex Differences at Baseline Conditions and in Response to Infection and/or Other Stimuli

Several models have been used to investigate sex differences at baseline conditions and in response to bacterial infection and/or other stimuli in the presence or absence of SP-A. Mikerov et al. (18) designed a number of studies using a model that involved exposure to filtered air or ozone prior to *Klebsiella pneumoniae* infection. In one study, there was an increased susceptibility to infection as depicted by decreased animal survival and phagocytic index of AMs in SP-A KO mice compared to WT. Although infected females showed a better survival than males, if the animals were exposed to ozone prior to infection females were more affected than males in both KO and WT mice (18). This was further described in a subsequent study where, in SP-A KO mice compared to WT, there was a decreased clearance of bacteria from the lung. Ozone-exposed females were more affected than males; however, in the absence of ozone-induced oxidative stress, males were more predisposed to have a higher level of dissemination of infection compared to females (19). In another study, via the use of a multianalyte immunoassay that measured 59 different cytokines, chemokines and other proteins, Mikerov et al. showed that wild type male mice exhibited a more exuberant response to infection alone and females exhibited a more robust response to infection with prior ozone exposure (20). Sex differences have been observed by histopathologic evaluation of lung and extrapulmonary tissues. In response to *K. pneumoniae* infection, more pronounced lesions were observed in extrapulmonary tissues (liver and spleen) in males compared to females. But if infection was preceded by ozone exposure (an oxidative stress), females exhibited a more excessive inflammatory response in lung than males (21). Sex differences in the AM proteome of KO and WT mice at baseline conditions (22, 23) showed that proteins such as the major vault protein, chaperonin subunit β CCT2 and Rho GDPα dissociation inhibitor, shown to interact with the estrogen receptor (24–26), were increased in females (22, 23).

Sex differences also have been observed after *K. pneumoniae* infection in gene expression in AM from KO and SP-A KO mice expressing an SP-A transgene (27). In the same type of mice (i.e., KO expressing an SP-A transgene), sex differences were observed: (1) in the AM (28–30) or type II epithelial cell (31) miRNome and in the miRNA-targeted genes after ozone exposure; (2) in AM cytoskeleton and cell morphology (32); (3) in the AM KO proteome after rescue with human SP-A variants at baseline conditions (33); and (4) in the BAL proteome in response to insults (34). A recent study by Xu et al. showed that AM from KO mice are more oxidized than AM from KO mice expressing an SP-A transgene after ozone exposure, as assessed by optical redox imaging analysis (35). No redox sex-dependent differences were observed in SP-A KO mice after ozone exposure, but in the presence of the SP-A transgene sex-specific differences were observed in the redox status with males exhibiting a higher mitochondrial reactive oxygen species (ROS) level (35). Furthermore, in a *K. pneumoniae* model, with or without subsequent methacholine, sex differences were observed in KO in certain airway function readouts (36). These together implicate sex as a variable and potentially a role for sex hormones.

Durrani et al. showed that gonadectomy of WT mice eliminated (females) or minimized (males) sex differences in survival after infection with or without ozone-exposure prior to infection. Moreover, sex-hormone treatment of gonadectomized mice resulted in a survival pattern similar to that of the non-gonadectomized WT (37). Gandhi et al. (38) explored in SP-A KO mice, the impact of ozone, sex, and gonadal hormones on BAL characteristics in infection with *K. pneumoniae*. In the infected KO mice, no sex differences were observed and after prior ozone exposure only limited sex differences at the later time points were observed in BAL parameters compared to WT under identical conditions. Furthermore, survival experiments with gonadectomized infected SP-A KO mice with or without prior ozone exposure showed no sex-specificity (38). This indicates that in the absence of SP-A and sex hormones, different mechanisms may be operative than those in WT mice that bring about sex-specific survival (18, 37). Of interest, in a model of *K. pneumoniae* infection, SP-A was found to bind to the Man α1 man sequence in the capsular polysaccharide of the K21a serotype of *K. pneumoniae* (39); this may be an important SP-A-mediated step in the process of providing host defense against *K. pneumoniae* infection.

## Viral Pathogens

Along with the extensive study of bacterial pathogens and SP-A, there have been numerous studies that investigated interactions of viral pathogens and SP-A. Distinct interactions have been identified with the Respiratory Syncytial Virus (RSV) F protein and SP-A (40). In an RSV infection model, SP-A KO mice compared to WT mice showed an increase in pulmonary infiltration, particularly PMNs after infection, an increased RSV burden in lung homogenates, an increase in the levels of pro-inflammatory cytokines, such as TNF-α and IL-6, and decreased superoxide and hydrogen peroxide generation by AMs. However, SP-A KO mice rescued with exogenous SP-A reduced viral titers and inflammatory cells in the lung (41). In an infection model with adenovirus, the SP-A KO mice compared to WT, showed a decreased clearance of adenovirus, increased lung inflammation and cytokines, and inflammatory cells in BAL, and a decreased uptake of adenovirus by AMs. Rescue by exogenous SP-A improved viral clearance and decreased inflammation and neutrophils in BAL (42).

Numerous studies have investigated interactions of SP-A with Influenza A virus (IAV). LeVine et al. showed that in SP-A KO mice compared to WT, there was a decreased clearance of IAV and an increase in pulmonary inflammation, whereas there was an increased viral clearance and decreased lung inflammation in exogenous SP-A-rescued KO mice. In addition, the SP-A-rescued mice showed a decreased myeloperoxidase activity in isolated PMNs from BAL, increased B and activated T lymphocytes in the lung and spleen, increased T helper (Th) 1 responses [interferon-γ, interleukin (IL)-2, and IgG2a] and decreased Th2 responses (IL-4, IL-10, and IgG1) in the lungs, 7 days after infection (43). Another study by Li et al. showed that in SP-A KO mice compared to WT after infection with a β-resistant strain of Influenza A virus, there was decreased survival and the mice had exaggerated early inflammation with increased MIP-2 protein levels and a greater influx of neutrophils (44). Hawgood et al. showed that rescue of SP-A KO mice with SP-A partially neutralized influenza A infection (39% neutralization) (45). A proposed mechanism for preventing viral dissemination was described for the influenza A infected mice. This involved binding of the sialylated asparagine 187 residue of SP-A to hemagglutinin that blocked access of neuraminidase to surface bound substrates (43). Furthermore, Watson et al. has reviewed the structural similarity of trimeric collectins of SP-A and SP-D with trimeric viral fusion proteins of RSV, IAV, and HIV and noted their therapeutic potential against infectious and inflammatory diseases (46).

## Other Infectious Agents

In an allergy model, following exposure to *Aspergillus fumigatus* antigen, it was shown that rescue of SP-A KO mice with SP-A, compared to KO controls, led to a decrease in peripheral eosinophilia and eosinophil peroxidase (EPO) activity and a decrease in IL-5, IL-2, and IL-10 on day 10. The ratio of interferon (IFN)-γ to IL-4 did not change significantly, but a decreased infiltration of eosinophils on lung histopathology on both days 4 and 10 after exposure was observed (47). Via the use of SP-A and inducible nitric oxide synthase (iNOS) double knockout mice it has been shown that lung injury and surfactant abnormalities observed after mycoplasma infection depend, in part, on these two molecules, SP-A and iNOS (48). Furthermore, a model with *Mycoplasma pneumoniae* infection showed SP-A to specifically bind the lipid A moiety of rough lipopolysaccharides desaturated phosphatidyl glycerol on the surface of *M. pneumoniae* (49). Another group demonstrated that mice lacking SP-A have increased airway hyperresponsiveness during *M. pneumoniae* infection vs. WT mice through TNF- α (50) or compared to KO expressing a specific human SP-A transgene (51). Furthermore, with this model it was shown that TNF-α activation of mast cells (MC) through the TNF receptor, but not the MC-derived TNF-α, lead to augmented airway hyperresponsiveness during *M. pneumoniae* infection when SP-A is absent. Moreover, *M. pneumoniae* infected KO mice engrafted with TNF-α negative or TNF receptor negative MC have decreased mucus production compared to mice with WT MCs. This work indicates a potential role of mast cells as secondary responders to TNF-α in host defense (52).

In an infection model with *Pneumocystis carinii*, SP-A-deficient mice compared to WT showed more severe infection and had an attenuated production of pro-inflammatory cytokines and reactive oxygen-nitrogen species (53). In a model of infection with *Pneumocystis murina*, although infection increased in both SP-A KO and WT mice over the study period, the overall intensity of the infection was more severe in SP-A KO mice indicating a protective role of SP-A (54). Both KO and WT cleared infection similarly but there were noted to be higher percentages of lymphocytes in SP-A KO mice and lower levels of IL-6 compared to WT, indicating that SP-A may modulate the immune response in this model (55).

## Surfactant-Related Activities

**Figure 1:**
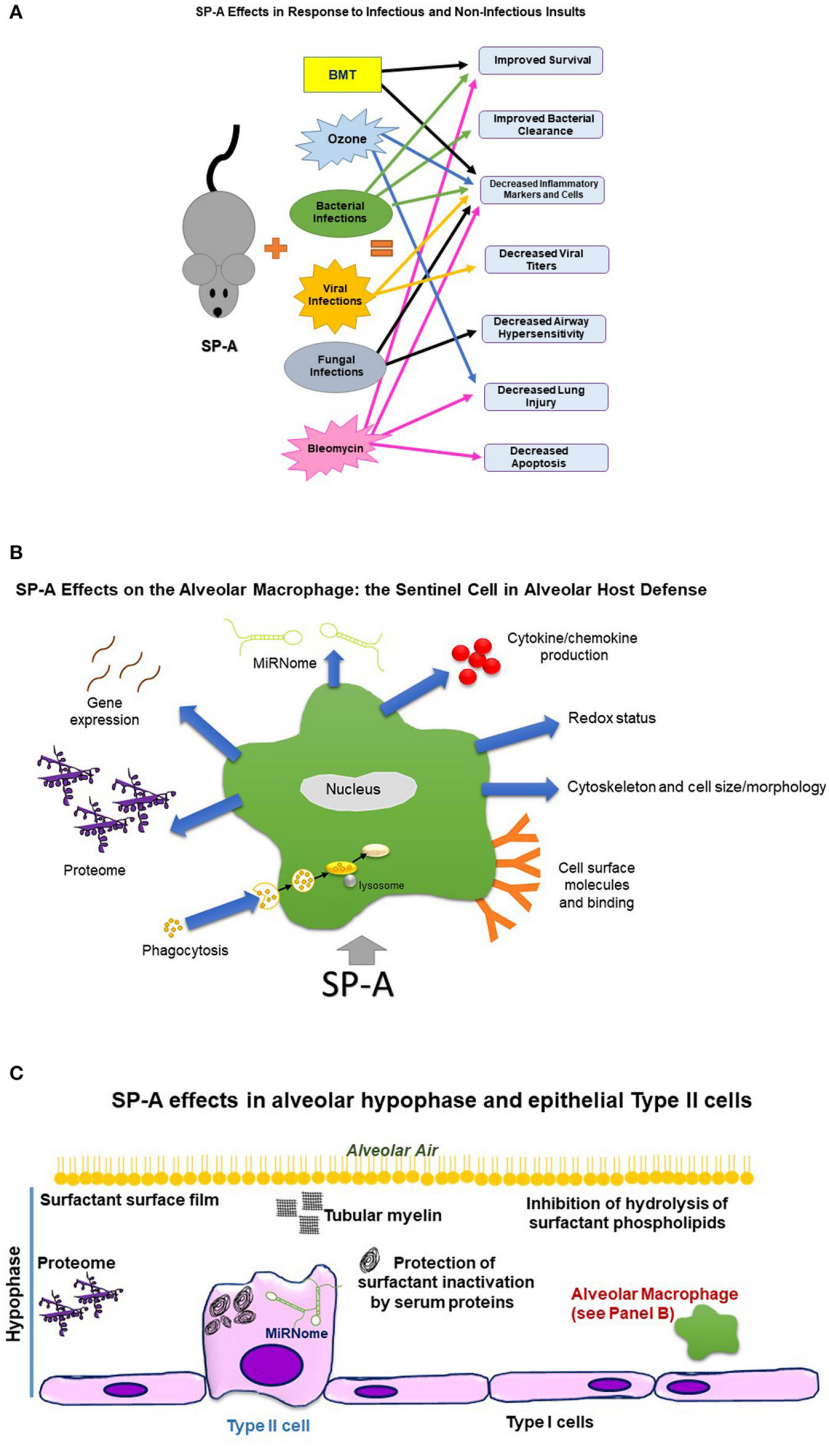
This figure depicts diagrammatically effects of SP-A on the organism, alveolar macrophage, and alveolar hypophase, as assessed by comparison of SP-A KO mice to WT or SP-A KO carrying a human SP-A transgene. **(A)** Depicts effects of SP-A on the organism in response to infectious and non-infectious insults. BMT, Bone marrow transplantation. Sex differences were observed in survival after bacterial infection, clearance of bacteria, dissemination of infection, robustness of response to infection, extrapulmonary lesions, and airway function readouts. SP-A in response to double insults, such as infection and ozone exposure, improved survival. **(B)** Depicts effects of SP-A on the alveolar macrophage, the sentinel alveolar host defense cell. Sex differences were observed in proteome, gene expression, miRNome, cytoskeleton, and redox status. **(C)** Depicts SP-A effects in the alveolar hypophase and epithelial Type II cells. Sex differences were observed in the miRNome of the epithelial Type II cells.

As a way of brief background, the alveolus, which is the distal lung air space, is lined by epithelial cells, the Type II and Type I cells. The former are the site for the production of surfactant; surfactant is essential in the prevention of alveolar lung collapse, and via this function enables the lung to carry out its vital function of O_2_/CO_2_ exchange. The latter, the Type I cells, are responsible for the O_2_/CO_2_ exchange, which is the key function of the lung and essential for life. The alveolar space is covered by a thin liquid layer, called hypophase, and the functioning surfactant monolayer is found at the air liquid interface. Under normal conditions the only immune cell found in the hypophase is the alveolar macrophage. Also, various morphological structures of surfactant such as tubular myelin, lamellar bodies and other, as well as proteins and other are found in the hypophase. A diagrammatic presentation of this is shown below in Figure 1C.

The effect of SP-A on various surfactant components has been evaluated in SP-A KO mice compared to WT. Alveolar and lung tissue saturated phosphatidylcholine pools were higher in KO, but the clearance of radiolabeled DPPC or SP-B from the air spaces after intratracheal injection of radiolabeled palmitic acid and choline was similar (56). Moreover, SP-A has been shown to be a necessary component for the formation of tubular myelin, an extracellular structural form of surfactant. KO mice lack tubular myelin (57, 58). However, if SP-A lacks its collagen-like region, tubular myelin is not restored in the KO mice, indicating a role for this SP-A region in tubular myelin formation. In addition, the absence of the SP-A collagen-like region results in functional defects of surfactant (59). Rescue of SP-A KO mice with surfactant from human bronchoalveolar lavage containing either both human SP-As (57) or expression of rat SP-A in Clara cells and alveolar type II cells of the SP-A KO mouse (59) restored tubular myelin forms.

Surfactant from KO mice exhibited a number of differences in its characteristics from that derived from wild type mice. These included, among others, a lower percentage of large surfactant aggregates (LA) and with cycling an increased conversion of LA (the biophysically active form of surfactant) to small vesicular forms, increased sensitivity to inactivation by serum proteins as assessed by measurements of minimum surface tension, surfactant with lower buoyant density, and other (58). Although it was previously reported that no differences between WT and KO were observed in the amount of LA (3), different methods were used. However, alterations in surfactant characteristics in KO mice compared to WT mice didn't seem to result in functional deficits, as assessed by exercise tolerance with swimming and running, and a 4-day period of hyperoxia did not affect survival and pressure-volume curves (60).

SP-A has been shown in *in vitro* studies to suppress synthesis of type IIA secreted phospholipases A2 (sPLA2-IIA) (61) as well as inhibit hydrolysis of surfactant phospholipids (62). The latter was shown to be the case *in vivo* following administration of sPLA2-IIA in mice. The SP-A KO mice exhibited a significantly higher level of surfactant phospholipid hydrolysis, indicating an SP-A inhibitory effect on this process and a potential role for SP-A in acute respiratory distress syndrome (ARDS), as changes in surfactant phospholipids become evident early in the course of ARDS. SP-A, via its ability to inhibit surfactant phospholipid hydrolysis and thus maintain, to a certain extent a well-functioning surfactant, plays a protective role in lung injury. This is of great relevance to the current Covid-19 pandemic, as sPLA2-IIA was shown to associate with markers more likely to lead to mortality after SARS-CoV-2 infection (63).

## Other Models

In a bleomycin-induced acute lung injury model, SP-A KO mice compared to WT, showed decreased survival, increased cytokine inflammatory response, lung edema, apoptosis, and lung injury. These observations indicate a role for SP-A in the regulation of inflammation, lung epithelial cell integrity, and apoptosis after non-infectious insults. Moreover, rescue with SP-A of SP-A KO bleomycin-treated mice improved survival to the level seen in WT (64).

In an ozone-induced oxidative stress model, SP-A KO mice compared to WT showed a larger increase in BAL total protein and an increase in LDH activity and phospholipid content. In WT mice there were more BAL PMNs and elevated MIP-2 and monocyte chemoattractant protein (MCP)-1 (65). In a model where exposure to *E. coli*-derived LPS combined with oxidative stress was studied, SP-A KO mice showed, in BAL, a lower number of cells at exposure to 0.5 ng of LPS but not at 2 ng of LPS, compared to WT, indicating an inability of KO to respond to low levels of LPS, but this inability was overcome in the face of a more severe insult (i.e., 2 ng LPS). After LPS and ozone exposure, KO showed a decrease in PMN recruitment, a decrease in MIP-2 levels, and an increase in phospholipids (66).

SP-A has also been shown to play a role in lung inflammation in a murine allogeneic bone marrow transplantation (BMT) model. SP-A KO mice after BMT showed increased inflammation along with decreased dynamic lung compliance and donor T-cell dependent mortality. However, treatment with SP-A improved survival and decreased inflammation in the BMT model (67). Furthermore, via the use of KO mice it was shown that SP-A is necessary for the protective effects of keratinocyte growth factor in terms of its ability to attenuate inflammation after BMT (68).

We postulate that SP-A functions by enhancing the efficiency of the alveolar macrophage in removing harmful substances or damaged cells that serve as pro-inflammatory stimuli. In the absence of SP-A these materials are not as readily cleared or neutralized, allowing these stimuli to persist and thus increasing the degree of inflammation. Thus, we contend that SP-A is not in itself anti-inflammatory, but its actions result in enhanced host defense function that ultimately lessens inflammation.

## Translational Work

A summary including findings related to SP-A in human health and disease have been previously published (69). Alterations in levels of SP-A have been reported in many acute and chronic lung conditions. In diseases in adults, changes in SP-A levels have been observed, among others, in acute respiratory distress syndrome (ARDS) (70, 71), asthma (72), chronic obstructive pulmonary disease (COPD) (73), idiopathic pulmonary fibrosis (74, 75), sarcoidosis and hypersensitivity pneumonitis (75, 76), and poor outcome and early lung transplant survival (77, 78). In neonatal populations altered SP-A levels have been observed in bronchopulmonary dysplasia (BPD) (79), children with cystic fibrosis (CF) (80), infants dying from respiratory distress syndrome (RDS) (81, 82) or severity of RDS (83). These observations have led to consideration in some cases for use of SP-A as a biomarker in clinical settings. SP-A has also been found in amniotic fluid, increasing in level from 32 weeks of gestation to term (84) and then decreasing at the time of labor (85). SP-A has a postulated role in clearance of pathogens from the amniotic fluid that is part of the proposed mechanism for the increased risk for infections at preterm gestational ages (84). It has also been proposed that SP-A secreted from the mouse fetal lung serves as a hormone in parturition, and experimental evidence supports an SP-A role in the initiation of parturition as well as a delay in its absence (86). There is also further mouse evidence that the complex cascade of interactions to signal parturition may involve SP-A and platelet activating factor (PAF) (87–89). In humans, SP-A was also shown to move from the placental amnion to the reflected amnion, indicating that SP-A may play a role in protecting the integrity of the amnion and the amniotic cavity from pro-inflammatory stimuli during pregnancy (90). There is also work to indicate a potential role of SP-A in preterm birth (91). These findings highlight the key roles SP-A plays in health and disease, as well as opportunities for continuation of work to translate findings from KO mice to the human.

## Summary and Comments

Research involving SP-A continues to reveal an increasing level of evidence regarding its critical role in host defense, surfactant-related functions, and other. Much has been learned by the study of SP-A KO mice. The effects of SP-A on the organism, alveolar macrophage and alveolar hypophase are diagrammatically depicted in Figures 1A–C, respectively. In general, SP-A KO mice are more susceptible to infection, whether bacterial or viral, as well as in response to other infectious and non-infectious insults. In addition, there is pathologic evidence of impaired defenses and ability to clear infection. Rescue of SP-A KO mice with exogenous SP-A has resulted in significantly improved survival and improved levels of markers of host defense or inflammation, underscoring further the importance of SP-A in host defense.

Although surfactant replacement therapy is used rather routinely in the clinical setting to treat prematurely born infants with respiratory distress syndrome, none of the widely used surfactant replacement preparations contains SP-A (1). Of interest, a major complication in prematurely born infants is infection. SP-A in animal studies is shown to be protective after infection as assessed by survival studies (18, 37, 38, 44, 92). Moreover, current experience indicates that surfactant treatment of babies with “simple” RDS or with infection is less efficacious (93, 94) or conclusions could not be reached for near-term or term infants with true or suspected bacterial infection (95). Thus, inclusion of SP-A in surfactant preparations (96) or usage by itself to treat not only premature infants, but individuals with a variety of conditions as suggested by the animal studies reviewed here warrants consideration. Moreover, the potential use of SP-A or functional fragments of SP-A for therapy (46, 72, 97) as it may relate to the current Covid-19 pandemic may be a plausible consideration. Potential scenarios for the role of SP-A in Covid-19 have been discussed elsewhere (35, 98, 99). Finally, sex differences, as observed in certain models, as well as the role of sex hormones in sex-specific outcomes, point to a continued need for further research and attention to the role of sex in study design and therapies.

## Author Contributions

LD reviewed the literature and wrote much of the manuscript. DP reviewed the literature and wrote parts of the manuscript. JF planned and oversaw the entire review and contributed to all aspects of the manuscript. All authors contributed to the article and approved the submitted version.

## Funding

This work was funded in part by the Evan Pugh Fund, the George Pedlow Fund, the John Ardell Pursley Memorial Research Fund, and the Center for Host Defense, Inflammation and Lung Disease (CHILD) Fund, Department of Pediatrics, Penn State College of Medicine.

## Conflict of Interest

The authors declare that the research was conducted in the absence of any commercial or financial relationships that could be construed as a potential conflict of interest.

## Publisher's Note

All claims expressed in this article are solely those of the authors and do not necessarily represent those of their affiliated organizations, or those of the publisher, the editors and the reviewers. Any product that may be evaluated in this article, or claim that may be made by its manufacturer, is not guaranteed or endorsed by the publisher.

## Abbreviations

AM: alveolar macrophage
ARDS: acute respiratory distress syndrome
BAL: bronchoalveolar lavage
BMT: bone marrow transplantation
KO: knockout
IAV: influenza A virus
IFN: interferon
IL: interleukin
iNOS: inducible nitric oxide synthase
LA: large aggregate
LPS: lipopolysaccharide
MC: mast cell
MIP: macrophage inflammatory protein
PMN: polymorphonuclear leukocyte
RDS: respiratory distress syndrome
ROS: reactive oxygen species
RSV: respiratory syncytial virus
SP-A, -B, -C, -D, surfactant protein-A, -B, -C: -D
sPLA2: secreted Phospholipases A2
TNF: tumor necrosis factor
TLR: Toll-like receptor
WT: wild type.
